# Longitudinal monoaminergic PET imaging of chronic proteasome inhibition in minipigs

**DOI:** 10.1038/s41598-018-34084-5

**Published:** 2018-10-24

**Authors:** Thea P. Lillethorup, Andreas N. Glud, Aage K. O. Alstrup, Ove Noer, Erik H. T. Nielsen, Anna C. Schacht, Natalie Landeck, Deniz Kirik, Dariusz Orlowski, Jens Christian H. Sørensen, Doris J. Doudet, Anne M. Landau

**Affiliations:** 1 Department of Nuclear Medicine and PET Center, Department of Clinical Medicine, Aarhus University and Hospital, Aarhus, Denmark; 2 Center for Experimental Neuroscience (CENSE), Department of Clinical Medicine, Aarhus University, Aarhus, Denmark; 3 Brain Repair and Imaging in Neural Systems (BRAINS) Unit, Department of Experimental Medical Science, Lund University, Lund, Sweden; 4 Department of Medicine/Neurology, University of British Columbia, Vancouver, BC Canada; 5 Translational Neuropsychiatry Unit, Department of Clinical Medicine, Aarhus University, Aarhus, Denmark

## Abstract

Impairment of the ubiquitin proteasome system has been implicated in Parkinson’s disease. We used positron emission tomography to investigate longitudinal effects of chronic intracerebroventricular exposure to the proteasome inhibitor lactacystin on monoaminergic projections and neuroinflammation. Göttingen minipigs were implanted in the cisterna magna with a catheter connected to a subcutaneous injection port. Minipigs were imaged at baseline and after cumulative doses of 200 and 400 μg lactacystin, respectively. Main radioligands included [^11^C]-DTBZ (vesicular monoamine transporter type 2) and [^11^C]-yohimbine (α2-adrenoceptor). [^11^C]-DASB (serotonin transporter) and [^11^C]-PK11195 (activated microglia) became available later in the study and we present their results in a smaller subset of animals for information purposes only. Striatal [^11^C]-DTBZ binding potentials decreased significantly by 16% after 200 μg compared to baseline, but the decrease was not sustained after 400 μg (n = 6). [^11^C]-yohimbine volume of distribution increased by 18–25% in the pons, grey matter and the thalamus after 200 μg, which persisted at 400 μg (n = 6). In the later subset of minipigs, we observed decreased [^11^C]-DASB (n = 5) and increased [^11^C]-PK11195 (n = 3) uptake after 200 μg. These changes may mimic monoaminergic changes and compensatory responses in early Parkinson’s disease.

## Introduction

Parkinson’s disease (PD) targets the motor system leading to resting tremor, bradykinesia and rigidity as the most characteristic symptoms. The onset of motor symptoms is preceded by a prodromal phase of several years with neurodegenerative processes and cellular changes that cause non-motor complications^1,2^. According to the Lewy body-based Braak’s pathology staging of PD, serotonergic (median raphe) and noradrenergic (locus coeruleus (LC)) nuclei located in the brainstem are affected in stage 2 of the disease, while dopaminergic neurons in the substantia nigra (SN) are affected later, in stages 3-4^3^. These changes in non-dopaminergic monoaminergic innervation are suspected to contribute significantly to the multiple non-motor symptoms of the disease, both in prodromal as well as in the manifested clinical condition^2,4^. In particular, depression and sleep disturbances, two major non-motor symptoms of PD, are known to involve considerable serotonergic and adrenergic components^5–7^. Defining the early monoaminergic alterations in early PD is therefore of crucial importance to understand etiology and pathophysiology and to evaluate therapeutic targets. Positron emission tomography (PET) imaging with specific tracers may provide the needed clues to identify patients at an early stage and to follow the status of these transmitters during disease progression.

The ubiquitin proteasome system (UPS) is the main intracellular pathway for protein degradation and its dysfunction has been implicated in the pathophysiology of PD^8^. The impairment of the UPS in PD is underpinned by the containment of alpha synuclein (α-syn), ubiquitin and the 20S proteasome catalytic core in Lewy inclusion bodies found in brains of PD patients^9,10^. Furthermore, post-mortem studies on patients with PD have reported decreased subunit expression and enzymatic activity of the proteasome in the SN compared to age-matched controls^11,12^. In order to investigate proteasome dysfunction as a model for PD, inhibitors of the UPS have been trialed using various administration routes. While peripheral administration leads to mixed effects, local nigral, medial forebrain bundle or striatal administration of UPS inhibitors consistently leads to dopamine deficits and PD symptomatology in rodent^13–18^. Here, we investigate the longitudinal effects of chronic direct intracerebroventricular (ICV) exposure to lactacystin on monoaminergic projections and, in a small subset, on neuroinflammation, using *in vivo* PET imaging and specific tracers. Due to its large gyrencephalic brain^19^, the Göttingen minipig is well suited for longitudinal and detailed *in vivo* imaging studies with multiple PET tracers^20–22^. Moreover, the large brain also facilitates the implant of chronic catheters and access ports.

To track and evaluate chemical deficits over time in response to ICV administration of the proteasome inhibitor, lactacystin, we used tracers of monoamine function and a validated tracer of inflammation. Evaluation of the dopaminergic and noradrenergic systems was done in 6 minipigs using (+)-α-[^11^C]-dihydrotetrabenazine ([^11^C]-DTBZ), a marker of vesicular monoamine transporter 2 (VMAT2) availability, routinely used in PD studies^23^, and [^11^C]-yohimbine, a marker of α_2_-adrenoceptors. [^11^C]-3-amino-4-(2-dimethylaminomethylphenylsulfanyl)-benzonitrile ([^11^C]-DASB), a marker of the serotonin transporter (SERT) was used for exploratory purposes in the last 5 minipigs. Finally, we also explored the neuroinflammatory component of the model using [*N*-methyl-^11^C](*R*)-1-(2-chlorophenyl)-*N*-(1-methylpropyl)-3-isoquinolinecarboxamide ([^11^C]-PK11195), a tracer of activated microglia, in a small group of 3 minipigs.

## Results

### Behaviour

**Figure 1:**
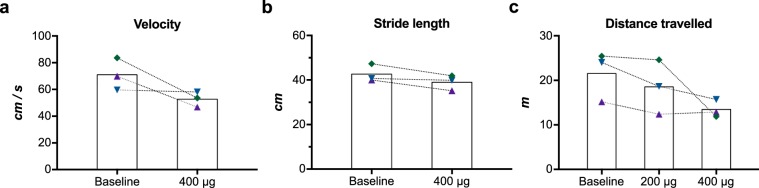
Behavioural assessment of the animals at baseline and after lactacystin injections. (**a**) Average velocity and (**b**) stride length were measured based on 4 quality readings for each minipig on a Gait mat. (**c**) Distance travelled was measured by videotracking the minipigs in their cages at baseline and after 200 μg and 400 μg lactacystin.

During the weekly visits to the minipig housing facility, decreased motility, dragging of feet along the floor, insecurity of the hind limbs when jumping for apples, narrow stance, crossing of hind limbs and freezing in place, were observed in all animals after only 3–4 injections of lactacystin (75–100 μg). In the three minipigs we were able to test quantitatively for behavioural abnormalities, an average of 24 ± 18% decreased velocity (%(post/baseline)-1) was recorded after a cumulative dose of 400 μg, compared to their own baseline values (Fig. 1a). However, the stride length of both hind and front limbs only mildly decreased from baseline values (Fig. 1b). Using EthoVision software, minipigs were also video monitored for 30 min at the same time of the day for 1 week at baseline and after 200 μg and 400 μg lactacystin. The distance travelled in their home pens was found to decrease by a mean of 15 ± 10% after 200 μg and by 34 ± 19% after 400 μg in the 3 minipigs compared to their own baseline recordings (Fig. 1c).

### [^11^C]-DTBZ PET

**Figure 2:**
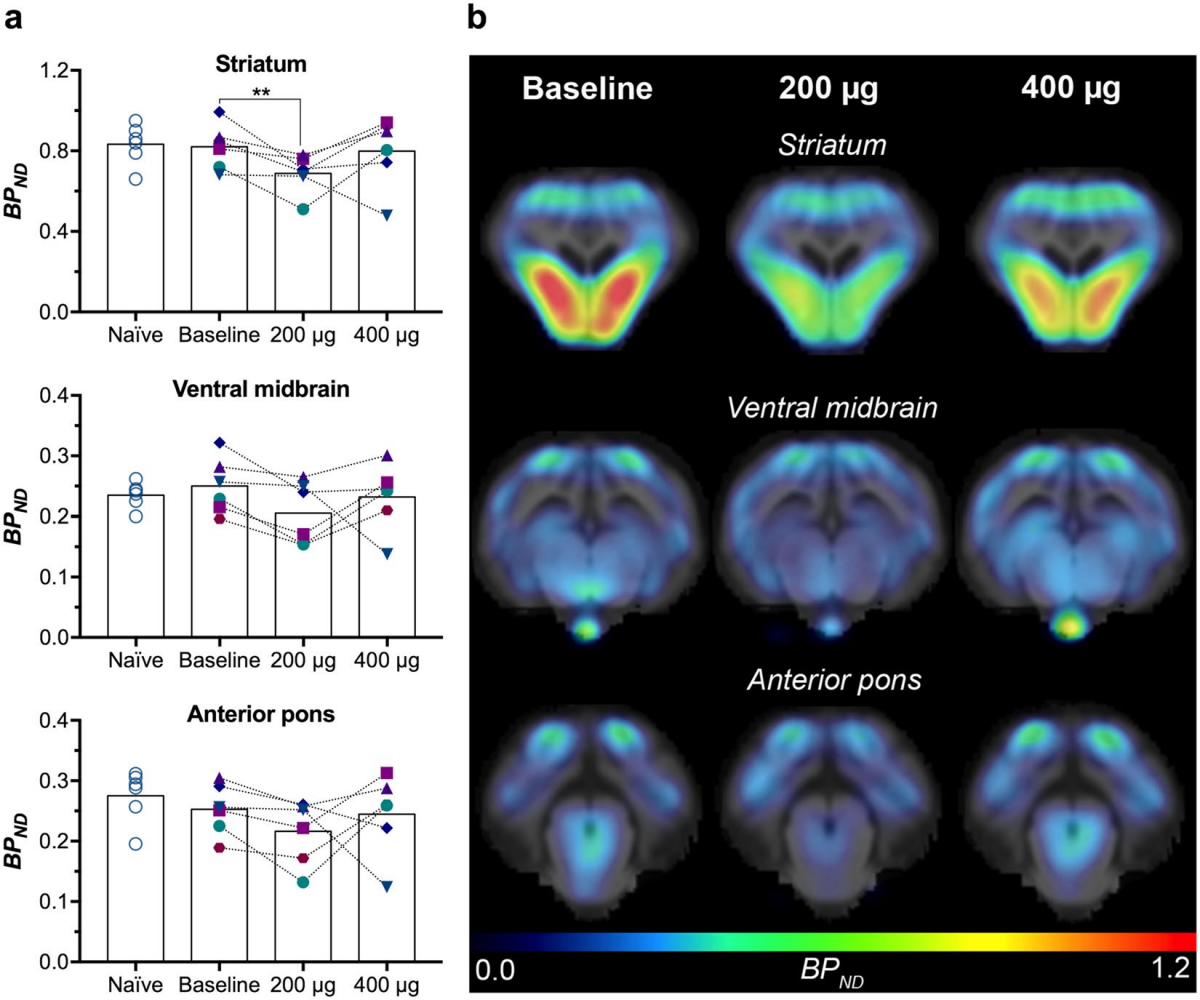
VMAT2 availability determined by *in vivo* [^11^C]-DTBZ PET. (**a**) [^11^C]-DTBZ *BP*_*ND*_ values calculated using Logan reference tissue model in striatum, ventral midbrain and anterior pons in 6 minipigs. Values are shown for naïve minipigs and for minipigs inserted with the injection port following a few saline injections (baseline), and after a cumulative dose of 200 and 400 μg lactacystin. **< 0.01 (**b**) *BP*_*ND*_ parametric maps are shown for a representative minipig at baseline, 200 and 400 μg in coronal view at the level of striatum, ventral midbrain and anterior pons.

Initially, we compared the [^11^C]-DTBZ baseline scans acquired after a few injections of saline with data from [^11^C]-DTBZ scans of naïve minipigs done as part of another project, to verify that ICV saline injections had no significant pharmacological effects (Fig. 2a). Using unpaired t-tests we found no statistically significant differences in striatum (0.83 ± 0.10 vs. 0.82 ± 0.11, mean ± standard deviation), ventral midbrain (0.24 ± 0.02 vs. 0.25 ± 0.05) or anterior pons (0.28 ± 0.04 vs. 0.25 ± 0.04). This indicated that neither the port implant nor the mechanical effect of injecting a small volume of liquid into the port had an effect on the [^11^C]-DTBZ binding potentials (*BP*_*ND*_) and confirmed the use of the scans acquired after a few injections of saline as appropriate baseline scans.

Next, using a two-way repeated measures ANOVA, regional [^11^C]-DTBZ *BP*_*ND*_ values of the minipigs at baseline were compared to those following a cumulative dose of 200 μg or 400 μg lactacystin (*n* = *6*). A significant effect of region (F(2, 15) = 138, *p* < 0.0001) and lactacystin dose (F(2, 30) = 4.7, *p* = 0.016) was found with no interaction between them (F(4, 30) = 0.97 *p* = 0.44). Dunnett’s multiple comparisons test of the effect of dose revealed a significantly decreased striatal binding potential of 16.1% (0.82 ± 0.11 vs. 0.69 ± 0.10, *p* = 0.007) after 200 μg lactacystin compared to baseline. Interestingly, *BP*_*ND*_ at the 400 μg dose was not significantly different from baseline. No statistically significant differences were observed in either the ventral midbrain or the anterior pons, though a trend towards decreased binding was observed in both regions after 200 μg lactacystin.

### [^11^C]-yohimbine PET

**Figure 3:**
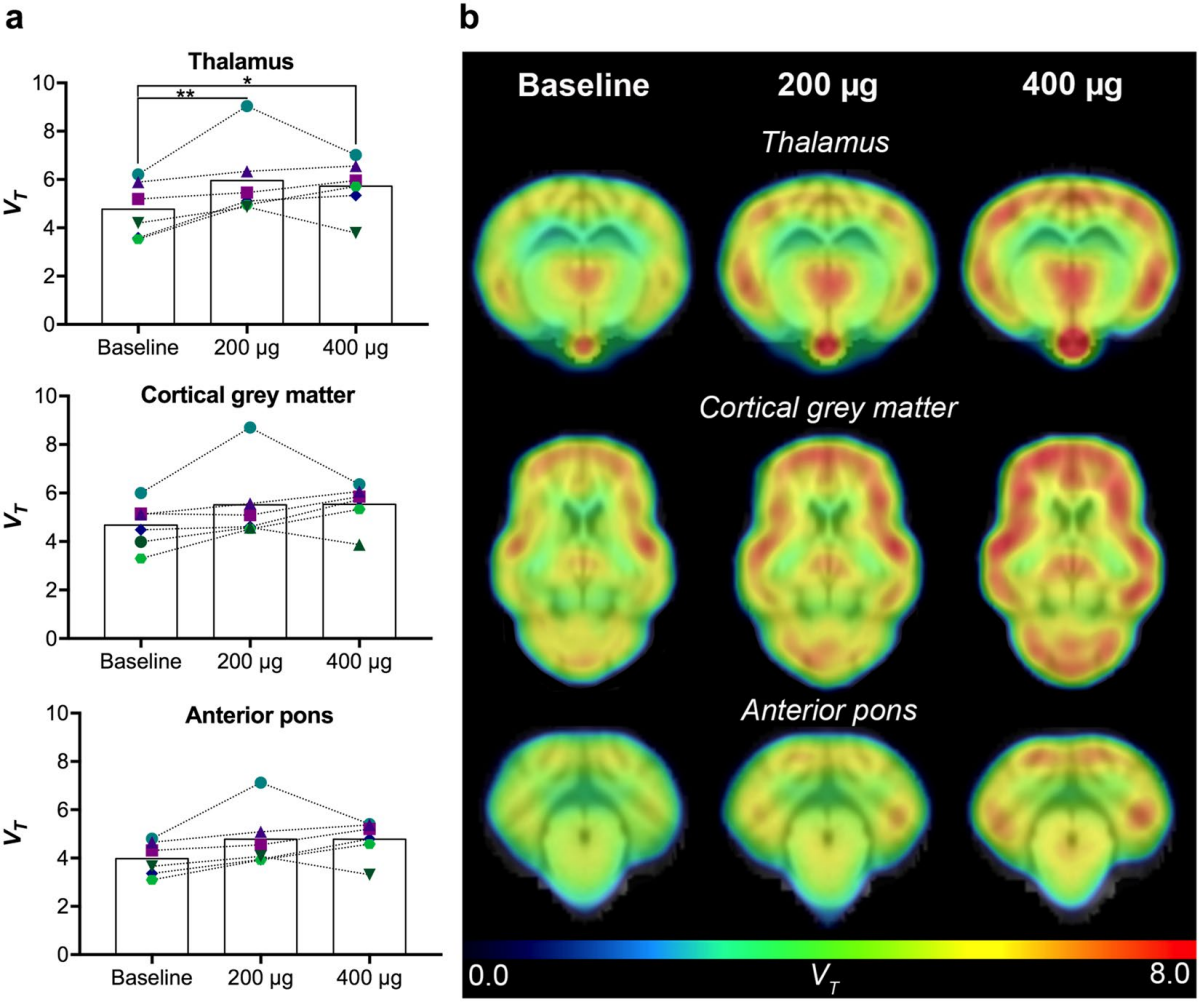
α2-adrenoceptor distribution determined by *in vivo* [^11^C]-yohimbine PET. (**a**) [^11^C]-yohimbine *V*_*T*_ values are displayed for thalamus, cortical grey matter and anterior pons after saline (baseline) and doses of 200 μg and 400 μg lactacystin in 6 minipigs. *< 0.05, **< 0.01 (**b**) *V*_*T*_ parametric maps are shown for a representative minipig at baseline, 200 and 400 μg in coronal view at the level of thalamus and anterior pons and transverse view for cortical grey matter.

We imaged the minipigs using [^11^C]-yohimbine PET, a highly selective antagonist of the α_2_-adrenoceptor at tracer doses, as a marker of noradrenergic neurotransmission^24^. Two-way repeated measures ANOVA analysis of the [^11^C]-yohimbine volume of distribution (*V*_*T*_) values (*n* = *6*) gave a significant effect of dose (F(2, 30) = 10.8, *p* = 0.0003) and no effect of region (F(2, 15) = 1.473, *p* = 0.26) or interaction (F(4, 30) = 0.157, *p* = 0.96). Dunnett’s multiple comparisons test revealed a significant increase in thalamus *V*_*T*_ values from baseline to 200 μg lactacystin (24.7%, 4.8 ± 1.2 vs. 6.0 ± 1.7, *p* = 0.009) (Fig. 3). A non-significant increase in *V*_*T*_ values was observed in cortical grey matter (18.3%, 4.7 ± 1.0 vs. 5.5 ± 1.7) and anterior pons (20.3%, 4.0 ± 0.7 vs. 4.8 ± 1.3) at the 200 μg dose compared to baseline. The increased binding persisted after 400 μg lactacystin and was significant in thalamus (19.2%, 4.8 ± 1.2 vs. 5.8 ± 1.2, *p* = 0.038) compared to baseline.

### Exploratory observations

#### [^11^C]-DASB PET

**Figure 4:**
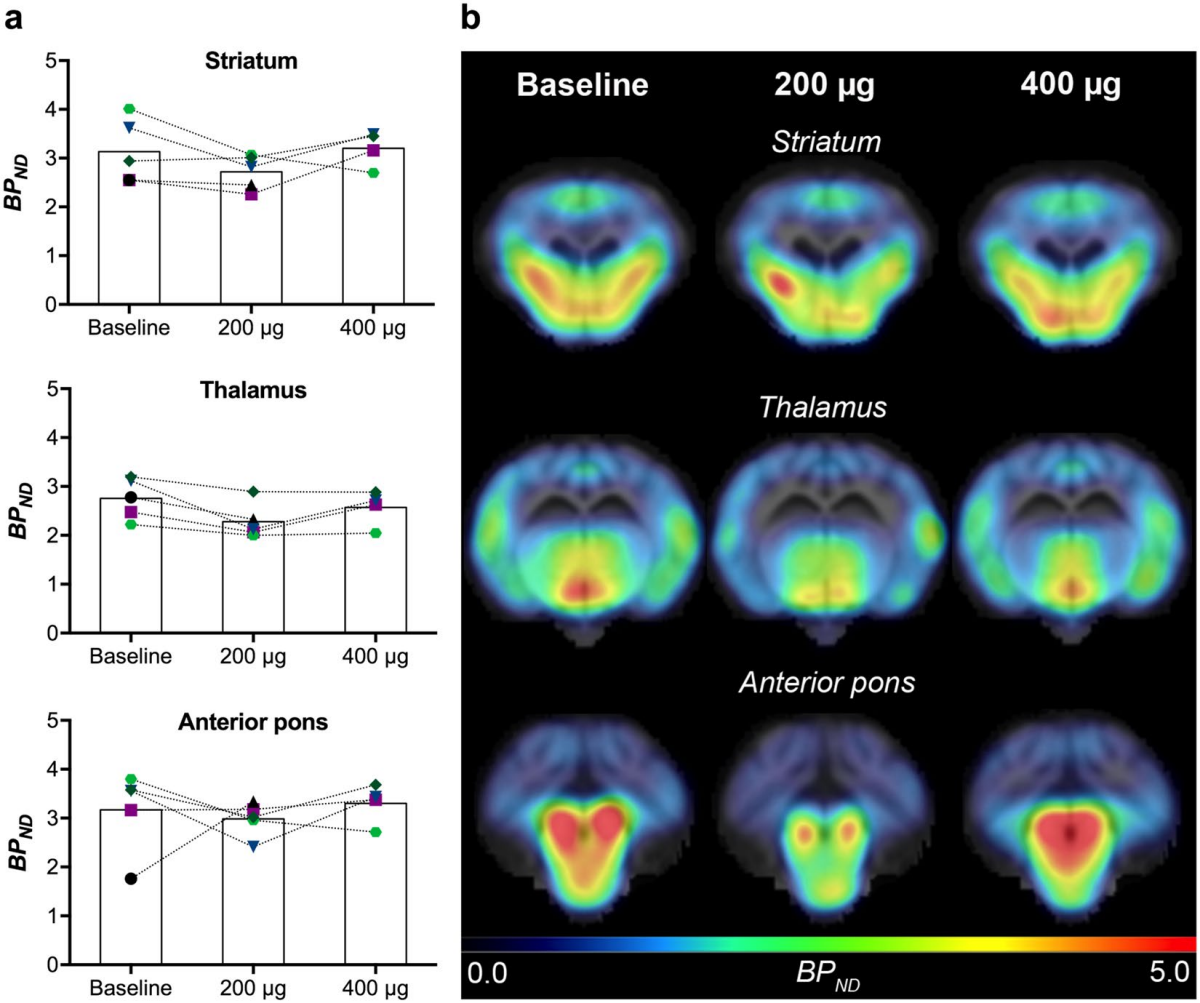
SERT distribution determined by *in vivo* [^11^C]-DASB PET. (**a**) [^11^C]-DASB *BP*_*ND*_ values are displayed for striatum, thalamus and anterior pons in 5 minipigs after saline (baseline) and doses of 200 μg and 400 μg lactacystin. (**b**) *BP*_*ND*_ parametric maps are shown for a representative minipig at the three different conditions in coronal view at the level of striatum, thalamus and the anterior pons.

Inspection of [^11^C]-DASB PET distribution in the smaller subset of animals (*n* = *5*) may suggest a trend towards decreased SERT *BP*_*ND*_ in the striatum (13.1%, 3.1 ± 0.7 vs. 2.7 ± 0.4), thalamus (18.2%, 2.8 ± 0.3 vs. 2.3 ± 0.3) and anterior pons (6.5%, 3.2 ± 0.8 vs. 3.0 ± 0.3) after 200 μg lactacystin compared to baseline. After 400 μg, no differences were observed compared to baseline (Fig. 4). Note that due to an error in the PET scan acquisition, we are missing data for the 400 μg scan in one of the animals.

#### [^11^C]-PK11195 PET

**Figure 5:**
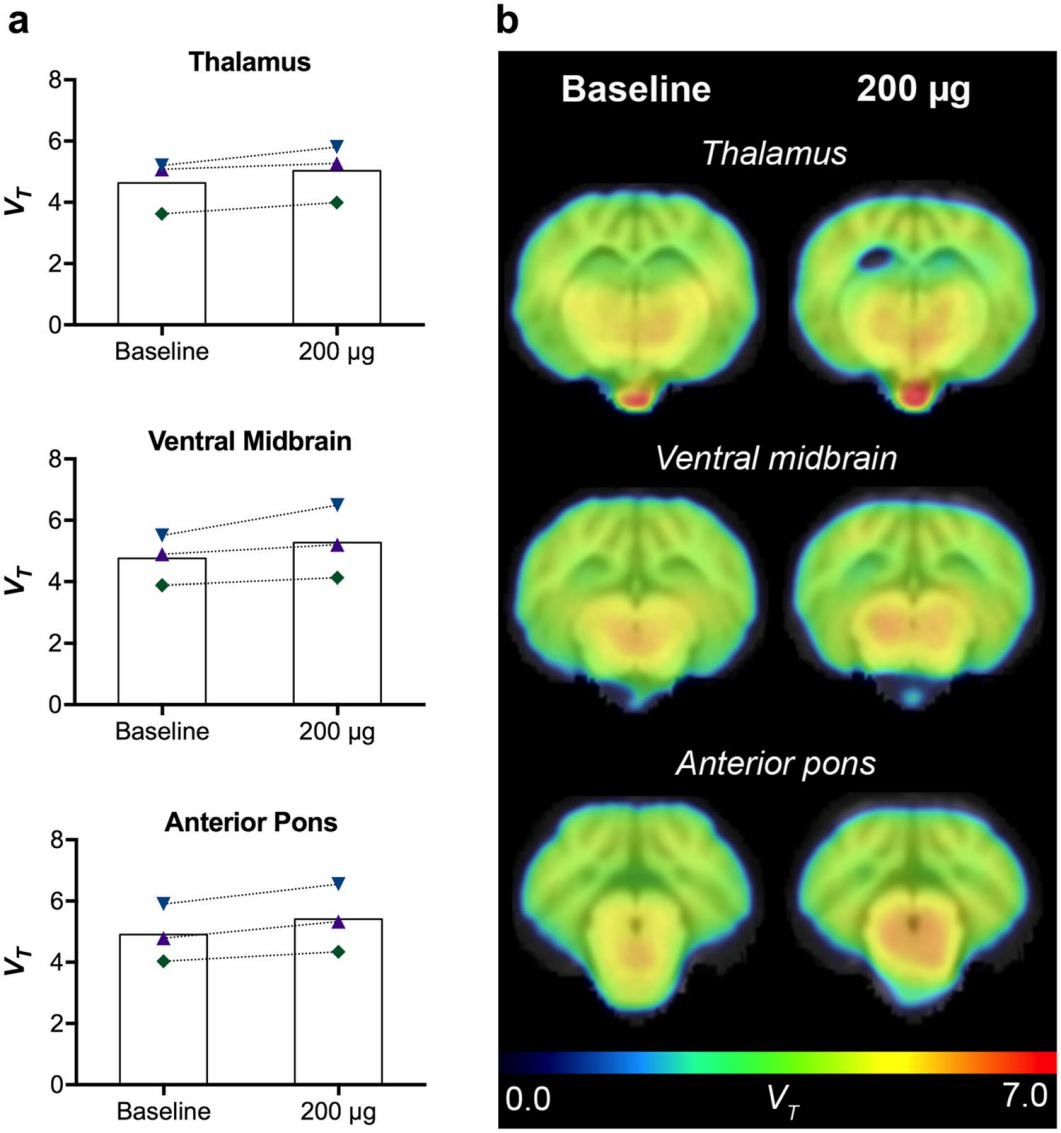
Microligal activation determined by *in vivo* [^11^C]-PK11195 PET. (**a**) [^11^C]-PK11195 *V*_*T*_ values are displayed for thalamus, ventral midbrain and anterior pons after saline (baseline) and a dose of 200 μg lactacystin. (**b**) *V*_*T*_ parametric maps are shown for a representative minipig at the baseline and 200 μg dose at coronal sections corresponding to the level of thalamus, ventral midbrain and anterior pons.

In order to evaluate the feasibility of tracking the potential inflammatory response to the lactacystin infusions in the last three minipigs, pilot scans with [^11^C]-PK11195 PET, a marker of activated microglia, were acquired at baseline and after 200 μg lactacystin, i.e. the dose that appeared to produce the maximal effect in earlier animals. In Fig. 5, the obtained *V*_*T*_ values are displayed in graphs. In thalamus, ventral midbrain and anterior pons, average increased binding of 8.4% (4.6 ± 0.9 vs. 5.0 ± 0.9), 10.8% (4.8 ± 0.8 vs. 5.3 ± 1.2) and 10.2% (4.9 ± 0.9 vs. 5.4 ± 1.1) were observed, respectively, which might indicate a mild, local inflammatory response.

#### CSF measurement of α-syn levels

When comparing CSF levels of α-syn in control minipigs (*n* = *3*) vs lactacystin-injected minipigs (*n* = *5*), we found no difference. The averages were 1250 pg/mL (±374) in the control vs 1056 pg/mL (±544) in the lactacystin-injected minipigs.

#### Immunohistochemistry

**Figure 6:**
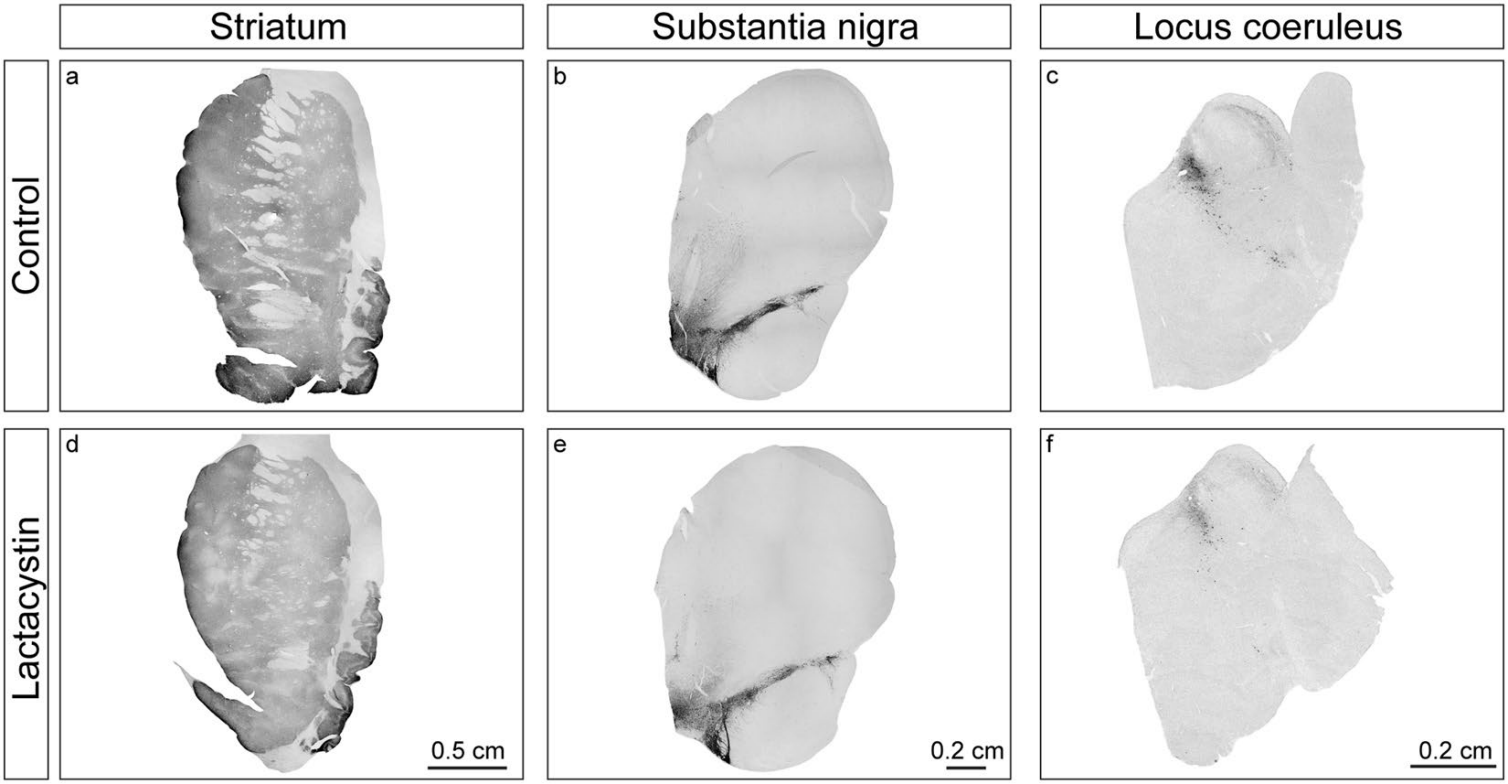
Preliminary immunohistochemistry of tyrosine hydroxylase positive fibers in the striatum (**a**,**d**) and neurons in the substantia nigra (**b**,**e**) and locus coeruleus (**c**,**f**) in a control minpig (top row) compared to a lactacystin-injected minipig (bottom row).

Tyrosine hydroxylase (TH) immunoreactivity is shown for one control and one lactacystin-injected minipig in Fig. 6. Visual inspection did not reveal differences at the level of the striatum (Fig. 6a,d) or SN (Fig. 6b,e) (*n* = *4*). For information purposes only, data are also presented for one lactacystin-injected minipig vs a control at the level of the LC (Fig. 6c,f).

## Discussion

In this proof of concept study, using multi-modal PET imaging, we were able to non-invasively track monoaminergic and inflammatory brain changes *in vivo* in Göttingen minipigs exposed to sustained UPS inhibition through a catheter in the cisterna magna coupled to a subcutaneous injection port. The purpose was to induce a model of PD slowly and progressively, which we hypothesized would be more akin to the situation in aging and PD as has been reported in other animals models^25–27^, and in contrast to the commonly used neurotoxin and lesion models focusing on dopaminergic degeneration. We observed changes in all three monoaminergic systems following repeated low dose intracisternal injections of the proteasome inhibitor lactacystin. In addition, a mild inflammatory response was detected.

Most studies of proteasome inhibition in animal models have been performed with intranigral^13,28^, intrastriatal^14,29^ or intra-medial forebrain bundle administration^30,31^, which in general have caused a direct and severe response in the animals investigated. *In vivo* studies in rats injected in the SN with high-dose lactacystin reported severe motor deficits, loss of [^11^C]-DTBZ binding in the striatum and a loss of TH in the SN^13,29^. In our recent study, we used the Göttingen minipig and injected lactacystin unilaterally into the medial forebrain bundle. We found a 36% decreased ipsilateral striatal [^11^C]-DTBZ binding accompanied by microglial activation, reduced SN TH-positive area, and decreased motor activity^31^.

In the current study, as opposed to the invasive intrastriatal or intranigral routes, we chose the ICV injection procedure to allow repeated administration and titration of lactacystin effects without further invasive manipulations of the animals. The synthetic, irreversible UPS inhibitor, lactacystin, was chosen since it is soluble in physiological saline without addition of DMSO or ethanol, solvents that can produce lesion effects of their own^32^. Compared with systemic injections, administration of lactacystin in the cerebrospinal fluid (CSF) might increase the bioavailability of the proteasome inhibitor, which was speculated to explain the reports of failed systemic administration^33,34^. Placement of a catheter in the cisterna magna is achievable in minipigs^35^ and through the medial aperture, circulation of CSF brings substances of interest in close proximity to the LC, median raphe and SN. The subcutaneous port thus allows for repeated delivery of the inhibitor to more systems including non-dopaminergic areas. Indeed, Matsui *et al*. injected lactacystin into the CSF of medaka fish and found a selective effect on dopaminergic and noradrenergic neuronal degeneration in addition to Lewy body formation and locomotor dysfunction^36^. Furthermore, in contrast to direct injections into the brain or lateral ventricles, which involves brief rupture of the blood-brain barrier and penetration of brain parenchyma, injections into the cisternal fluid are less disruptive. Thus, changes observed in the neurotransmitter systems, inflammatory response and behavioural analysis most likely stem from the ICV injected lactacystin instead of potential tissue damage caused by the needle injection. Indeed, our baseline [^11^C]-DTBZ data obtained after multiple ICV injections of saline are similar to the data obtained in naïve minipigs (Fig. 2a).

As expected from other animal studies and early PD cases^13,37^, we observed significantly decreased [^11^C]-DTBZ *BP*_*ND*_ to the VMAT2 in striatum, the major site of dopaminergic terminal density, and a similar trend in the ventral midbrain (site of dopaminergic cell bodies) and anterior pons (site of noradrenergic and serotonergic cell bodies) after repeated injections of lactacystin up to 200 μg. Similarly, in a small group of 5 minipigs, a decreased binding to presynaptic serotonergic terminals was observed in striatum (4/5 minipigs) and thalamus (5/5 minipigs) with [^11^C]-DASB after 200 μg lactacystin compared to baseline. However, interestingly the observed dopaminergic and serotonergic brain changes did not persist and, despite continued injections of lactacystin to a cumulative dose of 400 μg, were not different from baseline values.

DTBZ was initially suggested to be a stable marker of VMAT2 and to reflect dopamine terminal integrity in striatum^38,39^. Since then, its binding has been reported to be sensitive to vesicular dopamine fluctuations^40^. Thus, recovery of the striatal VMAT2 binding levels after 400 μg lactacystin could be explained by normalization of vesicular dopamine levels due to increased dopamine turnover and dopamine synthesis^41–44^, early compensatory responses to terminal damage. Previous studies suggest that PD may start with retrograde neurodegeneration, meaning that striatal dopamine terminals are impaired or lost prior to degeneration of the neurons located in SNc^45,46^. As the [^11^C]-DTBZ *BP*_*ND*_ values recovered and the TH stains were intact in both striatum and SN after 400 μg lactacystin, it appears that the initial decreases did not reflect terminal degeneration but rather transient impairment of the striatal synaptic function and vesicular dopamine transport. It is well known that compensatory mechanisms occur during the early and prodromal phases in order to slow the onset of the clinical symptoms of PD, which may include increased neuronal activity in the SN and increased dopamine turnover in the striatum^47,48^. These mechanisms have traditionally been difficult to study longitudinally in the clinical condition since most studies have been conducted after a diagnosis of PD. An advantage of the minipig model presented in the current study would be the ability to study pre-motor changes occurring early in PD.

[^11^C]-DASB is a reversible and highly selective ligand for SERT uptake. Widespread and symmetrical reduced presynaptic serotonergic function has been shown in striatum, midbrain, brainstem and cortical regions of both early and advanced idiopathic PD patients^49–51^. The mildly decreased [^11^C]-DASB binding in striatum, thalamus and anterior pons we detected after 200 μg lactacystin is thus consistent with some serotonergic impairment.

We propose the following hypotheses for the restoration of dopaminergic and serotonergic binding to near baseline values after a cumulative dose of 400 μg. First, the minipigs received the repeated injections of low dose lactacystin over up to 6 months, which could slowly have triggered compensatory mechanisms within the neurons that allowed them to cope with the impairment of the proteasome system. Indeed, it has been shown that proteasome inhibition can induce a compensatory activation of the autophagy-lysosomal pathway (ALP) and the UPS^52–55^, which would minimize the long term damaging effect of lactacystin on protein homeostasis. Together, the ALP and the UPS are responsible for degrading intracellular proteins, including α-syn, which is accumulated and aggregated in PD^56–58^. A study in transgenic mice has shown that, in healthy cells, the UPS is the main degradation pathway, however, during accumulation of α-syn, the ALP is recruited and the two systems complement each other^59^. Specifically, Ding *et al*. showed that chronic low-dose proteasome inhibition *in vitro* increased ALP and affected neural homeostasis, emphasizing the complex interplay between protein aggregation, proteasome activity and autophagy^53^.

Secondly, the neurons may have become insensitive to the low dose UPS inhibitor over time, which might explain the lack of sustained effect. Interestingly, sub-lethal doses of lactacystin can lead to a neuroprotective response by decreasing oxidative stress and increasing proteasome activity^60,61^. In addition, low dose proteasome inhibition significantly protected dopaminergic neurons against both 1-methyl-4-phenyl-1,2,3,6-tetrahydropyridine (MPTP) and 6-hydroxydopamine (6-OHDA) induced neurodegeneration, while forming α-syn and ubiquitin positive inclusions^62,63^. Even though we used doses of 20 μg, the compound was further dissolved in the CSF and might have been in too low concentrations at the site of the neurons to achieve a persistent effect over 6 months. However, as 200 μg lactacystin injected over a 3 month time period was sufficient to induce significant changes in [^11^C]-DTBZ and [^11^C]-yohimbine data, we did not choose to increase the dose further as our aim was not to induce an acute toxic effect, but instead, a slowly progressive effect. In retrospect, a higher dose should have been tested and would be of interest in future studies with the aim of obtaining persistent terminal degeneration. It is also possible that the compensatory, protective effect began sooner and that the deficit we observed at a dose of 200 μg only represents a fraction of what it may have been during earlier exposure. What is however consistent is that the response to low doses of lactacystin follows a U shape, an often classical physiological response to many injuries and pharmacological agents^64,65^.

The LC-noradrenergic system is one of the first brain neurotransmitter systems to show pathology in PD^3^. Pathology in LC leads to a diminished noradrenergic innervation and the levels of noradrenaline are markedly decreased in various brain regions in PD^66,67^. Indeed, a 20–30% reduction of noradrenergic function has been observed in the brains of PD patients^68^ and decline of noradrenergic projections has recently been confirmed *in vivo* by PET imaging using [^11^C]-MeNER, a marker of noradrenaline transporters in PD patients compared to controls^69^.

In this study we used [^11^C]-yohimbine, a selective marker of the α_2_-adrenoceptor with a wide brain distribution, and found a global increased *V*_*T*_ by 18–25% after 200 µg lactacystin in all the investigated brain regions compared to each animal’s own baseline, an increase which reached statistical significance in thalamus. In contrast to what was observed in the dopaminergic and serotonergic systems, the increased *V*_*T*_ of [^11^C]-yohimbine was sustained at a dose of 400 μg and remained significant in the thalamus compared to baseline. The increased [^11^C]-yohimbine *V*_*T*_ to α_2_-adrenoceptors likely reflects a general up-regulation of the α_2_-adrenoceptors in response to a loss of pontine noradrenergic cell bodies and/or lowered noradrenaline levels and may provide *in vivo* imaging evidence for early noradrenergic deficits corresponding to similar observations in PD patients^69^.

The sustained impairment of the noradrenergic system from lactacystin can in part be explained by its anatomical location. The catheter was inserted into the cisterna magna and the lactacystin-containing CSF would access the 4^th^ ventricle through the median aperture in close proximity to the LC. It has been shown that inhibition of the proteasome induced elevation of intracellular Ca^2+^ and cell death^70^. As Ca^2+^ influx can predispose to oxidative stress and the LC neurons express two Ca^2+^-type channels, it has been suggested that they are particularly vulnerable to Ca^2+^-triggered neurodegeneration in PD^71^ with less ability to recover. Interestingly, Matsui *et al*. observed a selective loss of dopaminergic and noradrenergic neurons following administration of lactacystin to the CSF^36^. As the LC neurons, similar to the SN neurons, contain pigmented neuromelanin and enzymes for catecholamine synthesis, it might increase their vulnerability to neurodegeneration^72^. Furthermore, it has been shown that reduced levels of VMAT2 lower the storage of catecholamines, which can lead to spontaneous neurodegeneration^73^.

Monoaminergic disturbances are associated with, and may even be the result of early inflammatory changes^74,75^. Activated microglia have been found in several brain areas of post-mortem PD tissue^76,77^ and in the brains of MPTP treated monkeys years after exposure to the toxin^78^. Here, in a small group of 3 minipigs, we performed an exploratory study to investigate the use of [^11^C]-PK11195 to follow inflammation. With limited access to the tracer, we chose to evaluate the 200 μg dose as we had already found the most consistent effect on the monoaminergic tracers at this dose in the earlier animals. As expected, we found an increased [^11^C]-PK11195 *V*_*T*_ of 8–11% at the 200 μg dose of lactacystin in thalamus, ventral midbrain and anterior pons, indicating a minor inflammatory response restricted to the midbrain. Previous studies using lactacystin in rodents have found activation of glial cells in the SN, indicating an underlying neuroinflammatory reaction as a response to proteasome inhibition^30,79^. While these results were obtained using injections directly into the nigrostriatal system, our mild response might be explained by low doses and route of administration, which avoids tissue damage from the needle injection.

Since this exploratory study was designed to assess longitudinal monoamine changes *in vivo*, a limitation is the lack of a thorough histological analysis. Minipigs were used as their own controls in order to reduce the number of animals in this longitudinal *in vivo* study, which did not give the possibility to access appropriate control tissue for post-mortem histological analysis. Despite this limitation, we managed to perform TH immunohistochemistry in a few of the animals and did not find any evidence of a dopaminergic cell loss in the SN or reduced fiber intensity in the striatum compared to one animal injected with only saline. Although there may be decreased TH immunoreactivity in the LC of the lactacystin-injected vs control minipig, this stain was only performed in one animal in each group and should be treated with caution. Also, although all animals were observed for behavioural changes during the weekly injections, these observations were subjective in nature. Unfortunately, not all animals could be tested quantitatively using the Gait mat and the activity tracking software as this equipment only became available after the study was already in progress. In the absence of saline-injected minipigs in parallel to the lactacystin-injected minipigs, we cannot exclude that the mild behavioral changes found after 200 and 400 μg lactacystin may simply represent normal changes in motor function over several months, i.e. an effect of aging and familiarity. Similarly, [^11^C]-DASB and [^11^C]-PK11195 became available to us later in the study and only a smaller subset of animals could be scanned. These data provide exploratory information with the caveat that the small number of animals does not permit statistical analysis.

This study provided, however, a wealth of information as to how to modify the design for increased efficacy, choice of tracers and behavioral analyses. For example, in future studies, we could progressively increase the dose of lactacystin over time to test whether this would maintain effectiveness of the inhibition and progression of the neurochemical losses and overcome the potential compensatory mechanisms. Alternatively, we could simultaneously inhibit both the UPS and ALP pathways to reduce the potential complementary actions. As both ALP and UPS decreases in activity are reported in both PD and aging, it may be necessary to inhibit the activity of both homeostatic systems to produce a progressive model of PD. Indeed, within the field of cancer research, it has been suggested that ALP automatically protects against and counteracts pro-apoptotic effects induced by preclinical therapeutic proteasome inhibition^80,81^.

This study demonstrates that repeated and controlled ICV administrations are feasible in a large animal model over several months. Furthermore, we show that the UPS inhibitor lactacystin induces early deficits in the dopaminergic, serotonergic and noradrenergic systems consistent with Braak staging of PD, and leads to presence of mild neuroinflammation. However, the transient changes in [^11^C]-DTBZ *BP*_*ND*_ and intact TH staining in the striatum and SN indicate that a higher dose of lactacystin should be tested in future studies to obtain a progressive model of PD. This model may be useful for studying the compensatory mechanisms in early disease and the debilitating non-motor symptoms of PD.

## Materials and Methods

### Animals

This study was approved and regulated by the Danish Animal Experiments Inspectorate (2012-15-2934-00074) and the experiments were carried out in accordance with the 2010/63/EU directive and the AARIVE guidelines. Eight adult female Göttingen minipigs (15 ± 4 months; 33 ± 10 kg) from Ellegaard Minipig Aps (Dalmose, Denmark) were housed in side-by-side cages (4.6 m^2^) where they had visual and snout contact with each other, at 20 °C and 50–55% relative humidity at Aarhus University animal facilities. The animals were kept on a restricted pellet diet and had free access to tap water. Each animal served as its own control to decrease variability in the imaging and behavioural experiments and reduce the number of animals required. Minipigs were acclimatized for one month prior to the surgeries.

### Surgery

Minipigs were pre-medicated intramuscularly with a mixture of 1 mg/kg midazolam and 6 mg/kg s-ketamine. A catheter (21G venflon) was inserted in an ear vein and anesthesia was induced using IV administration of 1 mg/kg midazolam and 3 mg/kg s-ketamine or 2 mg/kg propofol. Animals were intubated and mechanically ventilated using 2.1% isoflurane or 2–3% sevoflurane in 100–115 mL/kg/min 100% O_2_. Minipigs were placed in a MR-compatible stereotaxic frame developed specifically for pigs^82,83^. Local anesthetic (bupivacaine hydrochloride, 1 mg/kg, Marcaine®, AstraZeneca A/S) was injected subcutaneous at *ramus os zygomaticos* before fixating the skull to the frame and later at the planned incision site. The animals underwent a T1-weighted MR scan to identify the cisterna magna and local anatomy. Following the imaging procedure, temperature, reflexes, pulse and oxygen saturation were monitored and maintained during the surgery. The animals also received 0.03 mg/kg of buprenorphine hydrochloride IM (Temgesic®, Indivior UK Limited) on the morning and evening of the surgery day.

Using conventional sterile spine-neurosurgery methods, the occipital plane and the dura mater were exposed after carefully drilling away the bone over the cerebellum and dorsal to the cisterna magna. A polyurethane-catheter (3.5 Fr, Hydrocoat Catheter, Access Technologies Inc., USA) was inserted through a hole in the space between the brain and the dura mater and carefully descended into the cisterna magna. The patency was checked by slowly withdrawing a small amount of CSF. A small amount of BioGlue® (CryoLife, France) was used to secure the catheter to the skull at the entry hole. The catheter was then connected to a titanium injection port (Clearport Medium V-A-P, Access Technologies Inc., USA) which was then was fixed to the periosteum at the apex of the skull in a small subcutaneous pocket. Patency was ascertained again, withdrawing a small amount of CSF using a Huber needle inserted in the port and the catheter and port chamber were filled with sterile saline. Post-operatively, animals received approximately 500 mg pentrexyl or 750 mg cefuroxin on the morning and 30,000 IE/kg benzylpenicillin procain IM and 2 mg/kg flunixin oral once per day for 5 days following the surgery.

### Lactacystin injections

ICV injections were done under sterile conditions into the subcutaneous port every 7–11 days. For the procedure, the animals were sedated with 1.5 mg/kg of midazolam and 6 mg/kg of azaperone. The area surrounding the port was shaved and cleaned with 3 alternating washes of ethanol and iodine. A Huber point needle (22 ga, Access Technologies Inc., USA) was placed into the port and then connected to a 1 mL syringe under sterile conditions. No more than 0.6 mL solution (sterile saline or 20–40 μg lactacystin (CAS# 1258004-00-0, Calbiochem, Merck Millipore) dissolved in sterile saline) was injected into the port followed by up to 0.6 mL of sterile saline flush.

One month after the surgical port-insertion, sterile saline was injected into the port 2–3 times to check the patency of the setup and to ensure that there were no behavioural side effects from the injections into the port *per se*. No behavioral effects were found following saline injections. Following the completion of the baseline PET scans, 40 μg of lactacystin was injected into the port the first week. The dose was then lowered to 20 μg lactacystin every 7–11 days for the following weeks. The number of doses ranged from 16 to 22 in the different minipigs. PET scans were acquired at 2 time points after a cumulative dose of approximately 200 μg and 400 µg, corresponding to approximately 3 and 6 months of weekly injections.

### Behaviour

All minipigs were monitored for behavioural deficits during weekly visits to the minipig housing facility. In a later subset of minipigs, motor performance was assessed using a GAITfour® pressure mat connected to a gait analysis software. Minipigs (*n* = *3*) were tested at baseline, i.e. during the period of saline injections, prior to the first lactacystin administration, and following a cumulative dose of 400 μg lactacystin treatment. The minipigs were acclimatized to the pressure mat until a desired speed and pattern of their movements was acquired. The average of four quality readings from each minipig with minimum 3 consecutive gait cycles (all 4 hooves) was used for the software analysis to calculate the velocity and stride length of the limbs. Moreover, the same three minipigs were filmed by a camera installed above each home cage for 30 min at the same time of the day over a 1-week period at baseline, and after 200 and 400 μg lactacystin. EthoVision XT software (Noldus) was used to monitor and analyze the distance travelled.

### PET imaging

PET imaging was done at the PET Center at Aarhus University Hospital. Six to eight weeks after recovery from the catheter implant, and after a few sterile saline injections to verify port patency, minipigs were scanned at baseline with [^11^C]-DTBZ (*n* = *6*), a tracer of the VMAT2, [^11^C]-yohimbine (*n* = *6*), a marker of the α_2_-adrenoceptors, [^11^C]-DASB (*n* = *5*), a marker of SERT, and [^11^C]-PK11195 (*n* = *3*), a marker of activated microglia. Then minipigs underwent a series of lactacystin injections and were prepared for scanning after a cumulative dose of approximately 200 μg and 400 μg of lactacystin. Minipigs were prepared for PET imaging as described in Lillethorup, *et al*.^31^.

A computed tomography (CT) scan was obtained in a Biograph 64 Truepoint PET/CT scanner (6.7.2, Siemens) for attenuation correction. The average injected radiotracer dose and injected mass are listed in Table 1 for each scan. Radiotracers were dissolved in 10 mL saline for injection over 30 sec and the catheter was flushed with 10 mL saline. For all four tracers, 90 min PET data was reconstructed in 3D mode with a point-spread function (TrueX, 3 iterations and 21 subsets) and divided into 14 frames (5 × 60, 3 × 300, 4 × 600 and 2 × 900 sec). For PET scans with [^11^C]-PK11195, a catheter was placed in the femoral artery for blood sampling for the measurement of plasma radioactivity and parent fraction^84^. An average metabolite curve was constructed and used for all scans as previously reported^85^. Due to the femoral artery cut-down, animals received analgesics and antibiotics as needed.

**Table 1:** Radiotracer information.

	[^11^C]-DTBZ			[^11^C]-yohimbine			[^11^C]-DASB			[^11^C]-PK11195		
	ID	IM	N	ID	IM	N	ID	IM	N	ID	IM	N
Naïve	387 ± 14	2.1 ± 1.9	6									
Saline	364 ± 40	2.5 ± 3.6	6	378 ± 28	2.7 ± 5.5	6	401 ± 40	2.9 ± 4.0	5	371 ± 10	1.8 ± 0.8	3
200 μg	387 ± 34	0.6 ± 0.4	6	381 ± 34	1.4 ± 0.9	6	331 ± 47	3.4 ± 4.1	5	311 ± 125	2.6 ± 3.9	3
400 μg	384 ± 48	3.2 ± 3.1	6	375 ± 32	1.9 ± 1.4	6	344 ± 39	2.3 ± 2.4	4			

Average ± standard deviation of injected dose (ID, MBq) and injected mass (IM, μg) for each tracer. N is the sample size for each tracer at baseline and 200 and 400 μg doses of lactacystin.

### PET analysis

Reconstructed images were processed using PMOD v. 3.610 (PMOD Technologies, Switzerland) image analysis software. The images were rigidly co-registered to a MRI average of 22 minipig brains^86^. The acquired transformation was applied to the dynamic PET scan and volumes of interest were delineated using a minipig brain atlas^86^, including the striatum, thalamus, ventral midbrain (containing SN), anterior pons (containing LC and the median raphe) and a cortical grey matter mask. Time-activity curves were kinetically analyzed with different modeling approaches for each tracer. In order to reduce noise, large bilateral regions were used as no differences were found between the left and right hemispheres for any of the tracers.

For [^11^C]-DTBZ PET scans, the *BP*_*ND*_ was calculated at times of 20–90 min using Logan graphical analysis and for [^11^C]-DASB, the simplified reference tissue model was used. For both, the cerebellum excluding the vermis was used a region of non-displaceable binding. Because of the wide distribution of noradrenergic innervation throughout the brain, we could not identify a region of non-displaceable binding. Thus, [^11^C]-yohimbine was analyzed by computing the *V*_*T*_ using Logan analysis with a previously established population curve as input function corrected for weight and injected dose and omitting the first 20 min of each PET scan. As minipigs do not metabolize yohimbine peripherally, no correction was performed^24^. The population curve has previously been used and was based on 18 minipigs validated by comparing data using their own native curve and the population curve^20^. Sixty-minute dynamic time-activity curves from [^11^C]-PK11195 were kinetically analyzed using a 1-tissue compartment model with each minipig’s own measured plasma input to obtain the *V*_*T*_. Average parent fraction values fitted to a sigmoid model were used for all minipigs to correct the individual plasma input.

### Statistics

Statistical analysis of [^11^C]-DTBZ and [^11^C]-yohimbine PET were done using a two-way repeated measures ANOVA with factors being treatment and region and corrected using Dunnett’s post-hoc test. An unpaired t-test was used to compare [^11^C]-DTBZ data between naïve minipigs from a former study and the minipigs scanned at baseline after saline injections. In all analysis, *p* < 0.05 indicated significance. For the other two tracers and the behavioural data for which only 3 or 5 minipigs were included in the analyses, formal statistical analyses were not done; instead data is presented for each individual minipig.

### CSF α-syn measurements using the AlphaLISA platform

For the lumbar puncture, the minipig was placed prone on an operating room table with the hind limbs hanging over the side to facilitate kyphosis of the lumbar spine. Using a spinal needle (BD Spinal needle, Quincke, 20GA, 3.5 inch), a lumbar puncture was made between L4 and L5 or L5 and L6 vertebrae, under sterile conditions in deeply anesthetised minipigs (5 lactacystin-injected and 3 control) prior to perfusion. Crystal clear CSF was drained in a conical tube under steady drip-flow, no blood was noted in the samples. Samples were centrifuged and the supernatant was stored at −80 °C until further processing. α-syn levels were analyzed as described previously^87^. In brief, standard curves were established by 1:3 serially diluting serine 129 phosphorylated recombinant h-α-syn (MJFF, USA) into rat CSF (matrix). Unspiked rat CSF was used as blanks. Pig CSF samples were diluted 10:1 using a buffer containing 2% Triton X-100, 2% Tween 20 and 0.5 mg/ml BSA before each run. First, 5 μl of sample/standard protein was added into a 384-well OptiPlate (PerkinElmer, USA) in triplicates. A concentration of 50 μg/ml Eu Acceptor-beads coupled LB509 antibody (1:100 dilution from stock) and 5 nM biotinylated 4B12 antibody (1:100 dilution from stock) were mixed together beforehand in 1x assay buffer (10x AlphaLISA Immunoassay buffer, PerkinElmer, USA). After 1 h of incubation in the dark, 15 μl of Donor-beads (AlphaScreen® streptavidin-coated Donor-beads, PerkinElmer, USA) at a concentration of 66.7 μg/ml (1:75 dilution from stock) were added to each well and plate was incubated for 30 min in the dark. Then the plate was read using the EnVision 2104 Multilabel plate reader (PerkinElmer, USA) using the standard AlphaScreen emission filter (CW 570 nm). A relative standard deviation between sample and blank replicates of <11.4% was accepted.

### Immunohistochemistry

In a small group of lactacystin-injected minipigs, immunohistochemistry was performed with antibodies against TH at the levels of the striatum (*n* = *4*), SN (*n* = *4*) and LC (*n* = *1*), as previously described^31^ and compared to data from one control minipig.
